# The Methods of Obtaining Asepsis in Operations in Private Practice.—II

**Published:** 1898-08-20

**Authors:** W. McAdam Eccles

**Affiliations:** Assistant Surgeon to the West London Hospital, to the City of London Truss Society, &c.


### Medical Progress and Hospital Clinics.

[*The Editor will be glad to receive offers of co-operation and contributions from members of the profession. All letters should be addressed to* The Editor, at the Office, 28 & 29, Southampton Street, Strand, London, W.C.]

### THE METHODS OF OBTAINING ASEPSIS IN OPERATIONS IN PRIVATE PRACTICE.—II.

By W. McAdam Eccles, M.S.(Lond.), F.R.C.S.(Eng.), Assistant Surgeon to the West London Hospital, to the City of London Truss Society, &c.

In the former paper the preparation of the patient's skin in the operation area was discussed; now it will be well to look at the procedure to be followed in order to obtain a condition of the hands of the surgeon and his assistants which will not lead to an infection of the wound which is to be made in an aseptic area.

All who attempt operative surgery, or take any part in the same, must pay the utmost attention to the cleansing of their hands and forearms.

The nails should be always kept short. Before the operation the surgeon, and likewise every assistant, including the nurse, who has to undertake any duty bringing them into direct contact with the operation region, is to perform a thorough washing of the hands and forearms. Hot water and soap are of the greatest utility, and these, combined with the vigorous application of a sterile nailbrush, are of paramount importance in the cleansing of these parts.

It is necessary to allude to the fact that it often occurs that the nailbrush employed is in reality worse than useless, as it is the haven of multitudes of bacteria. Every surgeon should have his own brush, which he carries with him for the use of himself and his assistants, and which brush is sterilised after every operation and kept in a glass receptacle, which is only opened at the time of the cleansing. After the hands and adjacent parts have been thus scrubbed, no towel should be used. This detail would appear to be almost so small a one that it should not be mentioned, but it is a fact that a septic towel is not very infrequently applied to hands which should be considered far too clean for such.

Therefore, the hands are to be taken straight after the washing to the bowl of antiseptic solution. This should contain either biniodide or perchloride of mercury in the proportion of one part of the salt to a thousand parts of water. The hands and forearms are to be kept immersed in the antiseptic for at least five minutes. It is essential that they do not come in contact with anything that is septic before the actual operation is commenced, for such is very easy to occur unless the surgeon is constantly on his guard against it. For instance, he must not help in the arranging of the patient, or of the clothes, for if he do so he must again cleanse his hands as already described.

It is always to be remembered, in connection with the preparation of the hands of the surgeon and his assistants, that all that can be accomplished is to clean the surface of the skin, for in the short space of time at their disposal it is impossible that the depths of the integument can be reached. However, this is not an essential, for it is altogether a different matter from the purifying of the actual operation area in the patient. The surgeon has to bring merely the superficial aseptic portion of his skin in contact with the wound, but his knife has to traverse the depths of the integument of the patient in order to make that wound. In fact, there is much less likelihood of the surgeon's hands infecting the incision, if they have been washed as directed, than of its being rendered septic from the glands in the depths of the patient's skin.

The actual instruments used in the operation need to be as thoroughly aseptic as the skin surface. Most, if not all the instruments employed at the present time are wholly made of metal, and thus they can be rendered sterile by boiling in water containing a small amount of carbonate of soda to prevent rusting, provided that such instruments have been plated. If the instruments cannot be boiled immediately prior to the operation, it will be needful to wrap them after the boiling in some aseptic gauze, and remove this only at the moment when they are put into a tray containing a solution of carbolic acid, a solution of the strength of one part of the acid to forty parts of water.

If any instrument cannot from its nature be boiled without causing it harm, then it is essential that it should be thoroughly scrubbed with soap and water, and afterwards treated with strong solution of carbolic acid, say one in ten. Heat is, however, the surest means to render the instruments aseptic. With regard to sponges, these had better be entirely discarded, and their place taken by antiseptic gauze pads, or wool wrung out of an antiseptic solution.

The whole operation region should be surrounded with towels which have been soaked in, and wrung out from, a solution of either the perchloride of mercury or carbolic acid.

If the above methods be employed in cases where time will allow of it, there is little doubt that asepsis, or at any rate a condition which is so near it that for all intents it is asepsis, will be obtained, and healing will occur without fear of suppuration.

In cases, on the other hand, in which there is not time enough to permit of a thorough cleansing as above indicated, freedom from sepsis is not by any means so certain. The line of practice to be followed in such instances is to wash the parts with soap and water, using as much of the scrubbing process as the nature of the lesion will allow, at the same time shaving the region. This is to be succeeded by a liberal application of turpentine or ether, after which, the excess having been removed, an antiseptic solution stronger than that described before is to be employed. If any time has to elapse subsequent to this cleansing, then an antiseptic dressing should be kept over the part until the actual operation is performed.

This procedure should never be neglected when there is a probability that an operation will have to be performed within a short space of time, for if it be not undertaken then there will of necessity be a greater chance of sepsis resulting than if the simple precaution indicated is carried out. It must be confessed that in private practice these emergency cases do not cause nearly so much anxiety as do those that have to be dealt with in hospital work, provided that an attempt to obtain asepsis is really made, but if it is not, then such cases are certainly in a worse plight than the hospital patient.

